# Dorsal-Ventral Differences in Modulation of Synaptic Transmission in the Hippocampus

**DOI:** 10.3389/fnsyn.2020.00024

**Published:** 2020-06-18

**Authors:** George Trompoukis, Costas Papatheodoropoulos

**Affiliations:** Laboratory of Physiology, Department of Medicine, University of Patras, Patras, Greece

**Keywords:** hippocampus, dorsal-ventral, synaptic transmission, heterosynaptic modulation, GABA_B_ receptor, A1 adenosine receptor, GABA_A_ receptor, ion channel

## Abstract

Functional diversification along the longitudinal axis of the hippocampus is a rapidly growing concept. Modulation of synaptic transmission by neurotransmitter receptors may importantly contribute to specialization of local intrinsic network function along the hippocampus. In the present study, using transverse slices from the dorsal and the ventral hippocampus of adult rats and recordings of evoked field postsynaptic excitatory potentials (fEPSPs) from the CA1 stratum radiatum, we aimed to compare modulation of synaptic transmission between the dorsal and the ventral hippocampus. We found that transient heterosynaptic depression (tHSD, <2 s), a physiologically relevant phenomenon of regulation of excitatory synaptic transmission induced by paired stimulation of two independent inputs to stratum radiatum of CA1 field, has an increased magnitude and duration in the ventral hippocampus, presumably contributing to increased input segregation in this segment of the hippocampus. GABA_B_ receptors, GABA_A_ receptors, adenosine A1 receptors and L-type voltage-gated calcium channels appear to contribute differently to tHSD in the two hippocampal segments; GABA_B_Rs play a predominant role in the ventral hippocampus while both GABA_B_Rs and A1Rs play important roles in the dorsal hippocampus. Activation of GABA_B_ receptors by an exogenous agonist, baclofen, robustly and reversibly modulated both the initial fast and the late slow components of excitatory synaptic transmission, expressed by the fEPSPslope and fEPSP decay time constant (fEPSP_τ_), respectively. Specifically, baclofen suppressed fEPSP slope more in the ventral than in the dorsal hippocampus and enhanced fEPSP_τ_ more in the dorsal than in the ventral hippocampus. Also, baclofen enhanced paired-pulse facilitation in the two hippocampal segments similarly. Blockade of GABA_B_ receptors did not affect basal paired-pulse facilitation in either hippocampal segment. We propose that the revealed dorsal-ventral differences in modulation of synaptic transmission may provide a means for specialization of information processing in the local neuronal circuits, thereby significantly contributing to diversifying neuronal network functioning along the dorsal-ventral axis of hippocampus.

## Introduction

Hippocampus is an elongated prototypical brain structure thought to be composed of the repetition of a basic neural circuit of excitatory synaptic connections transversely positioned to the longitudinal axis of the structure, which in rodents is extended from a septal location, dorsally, to the temporal lobe, ventrally. Hippocampus has been found to be implicated in several brain functions which, however, are heterogeneously distributed along the longitudinal axis of the structure (Small et al., 2011; Strange et al., 2014). According to a current consensus, the role played by the most dorsally located hippocampal segment is on cognitive operations like spatial navigation, while internally monitoring functions related to emotionality are taken on by the ventral segment of the hippocampus (Bannerman et al., 2014; Strange et al., 2014). Though this functional segregation along the dorsal-ventral hippocampal axis might be considerably supported by a differentiated pattern of extrahippocampal connections of consecutive hippocampal segments, it nevertheless poses an important issue about the role that the endogenous neuronal circuitry may play in this uneven distribution of functions. Indeed, a growing body of recently acquired experimental evidence indicates that the endogenous network of the hippocampus is diversified along the long axis of the structure according to several aspects of organization, including gene expression patterns, intrinsic properties of principal cells and properties of synaptic plasticity (Papatheodoropoulos and Kostopoulos, 2000a; Maruki et al., 2001; Dong et al., 2009; Dougherty et al., 2012; Honigsperger et al., 2015; Cembrowski et al., 2016; Kouvaros and Papatheodoropoulos, 2016b; Malik et al., 2016; Milior et al., 2016; Schreurs et al., 2017; Floriou-Servou et al., 2018; Manahan-Vaughan, 2019); recently reviewed (Papatheodoropoulos, 2018). These data suggest that specializations in the endogenous hippocampal circuitry may fundamentally support functional segregation which is observed at a higher level of organization. Accordingly, the revealing of mechanisms of intrinsic network diversification along the dorsal-ventral hippocampal axis is a major challenge in the field of hippocampus research.

Among the most fundamental mechanisms that can functionally diversify local neuronal networks is the modulation of synaptic transmission (Giocomo and Hasselmo, 2007; Dayan, 2012; Marder, 2012; McCormick and Nusbaum, 2014). In the hippocampus, particularly interesting forms of regulation of excitatory synaptic transmission with important physiological implication are those phenomena of short-lasting heterosynaptic plasticity, lasting from seconds to minutes, collectively called heterosynaptic depression (Isaacson et al., 1993; Vogt and Nicoll, 1999; Molyneaux and Hasselmo, 2002; Serrano et al., 2006; Andersson et al., 2007; Covelo and Araque, 2016). Experimentally, heterosynaptic depression is manifested as a suppression of glutamatergic excitatory synaptic transmission in inactive synapses induced by strong stimulation of a different synaptic input into the same neuronal population (Covelo and Araque, 2016). Heterosynaptic depression is a complex phenomenon encompassing different forms of suppression of excitatory transmission, distinguished between each other based on time-course and underlying mechanisms. Short-lasting heterosynaptic depression lasting from milliseconds to a few seconds can be induced by short bursts of high-frequency stimulation (Isaacson et al., 1993; Zhang et al., 2003; Andersson et al., 2007), while induction of heterosynaptic depression that lasts up to several minutes requires long trains of high-frequency stimulation (Grover and Teyler, 1993a; Manzoni et al., 1994; Serrano et al., 2006). Given the physiologically important role that heterosynaptic depression can play (Dunwiddie and Lynch, 1978; Frerking and Ohliger-Frerking, 2006) it is especially interesting to examine this phenomenon comparatively between the dorsal and the ventral hippocampus.

In principle, the mechanisms that control the release of transmitter via activation of receptors sited at presynaptic terminals (Wu and Saggau, 1997; Miller, 1998; Frerking and Wondolowski, 2008) play pivotal roles in regulating synaptic transmission and the balance between excitation and inhibition, thereby crucially modulating local neuronal circuit function (Giocomo and Hasselmo, 2007; Dayan, 2012). In the hippocampus several neurotransmitter receptors regulate the synaptic release of transmitters (Thompson et al., 1993; Wu and Saggau, 1997; Miller, 1998). Among these receptors GABA_B_ receptor (GABA_B_R) is a key regulator of excitatory and inhibitory transmitter release in the hippocampus (Vizi and Kiss, 1998; Ulrich and Bettler, 2007). GABA_B_Rs have a broad distribution in the hippocampus (Bowery et al., 1987) and recent immunohistochemical data show that GABA_B_Rs in the apical dendrites of CA1 pyramidal cells are more abundant in the ventral than in the dorsal hippocampus (Dubovyk and Manahan-Vaughan, 2018). However, the role of GABA_B_Rs in controlling excitatory synaptic transmission in the dorsal and the ventral hippocampus has never examined before.

In the present study, we aimed to compare forms of regulation of synaptic transmission between the dorsal and the ventral CA1 hippocampal field, using two experimental approaches. We studied transient heterosynaptic depression (tHSD) comparatively in the two hippocampal segments and we found a stronger heterosynaptic effect in the ventral compared with the dorsal hippocampus. Several mechanisms appeared to contribute to tHSD, including GABA_B_Rs, GABA_A_ receptors, adenosine A1 receptors, and L-type voltage-gated calcium channels (L-VGCCs). Furthermore, these mechanisms contribute differently to t-HSD in the dorsal and the ventral hippocampus. We also examined the effects of GABA_B_R activation by an exogenous agonist, baclofen, and we found that baclofen suppressed the initial fast component of excitatory synaptic transmission more in the ventral than in the dorsal hippocampus and enhanced the late slow component of excitatory transmission more in the dorsal than in the ventral hippocampus. Finally, exogenous activation of GABA_B_Rs produced a similar enhancement of paired-pulse facilitation in the two segments of the hippocampus. Possible implications of these dorsal-ventral differences are discussed.

## Materials and Methods

### Animals and Slice Preparation

One hundred and eighteen adult male Wistar rats (RRID: RGD_10028) were used in this study. Animals were maintained at the Laboratory of Experimental Animals of the Department of Medicine, University of Patras (license No: EL-13-BIOexp-04), under controlled conditions of light-dark cycle (12/12 h) and temperature (20−22^o^C), and they had free access to food and water. All animal treatment and experimental procedures were conducted in accordance with the European Communities Council Directive Guidelines for the care and use of Laboratory animals (2010/63/EU − European Commission) and they have been approved by the “Protocol Evaluation Committee” of the Department of Medicine of the University of Patras and the Directorate of Veterinary Services of the Achaia Prefecture of Western Greece Region (reg. number: 187531/626, 26/06/2018). Thin slices from the dorsal and the ventral hippocampus were prepared as previously described (Papatheodoropoulos and Kostopoulos, 2000a; Kouvaros and Papatheodoropoulos, 2016b). Specifically, rats were sacrificed by decapitation under deep anaesthesia with diethyl-ether, then the brain was removed from the cranium and placed in ice-cold (2−4^o^C) standard artificial cerebrospinal fluid (ACSF) containing, in mM: 124 NaCl, 4 KCl, 2 CaCl_2,_ 2 MgSO_4,_ 26 NaHCO_3_, 1.25 NaH_2_PO_4_ and 10 glucose. ACSF was equilibrated with 95% O_2_ and 5% CO_2_ gas mixture at a pH = 7.4. The hippocampus was excised free from the brain and transverse 500 μm-thick slices were prepared from the dorsal and ventral hippocampus extending between 0.5 and 4.0 mm from each end of the longitudinal structure of hippocampus using a McIlwain tissue chopper. Immediately after their preparation slices were transferred to an interface type recording chamber where they were continuously perfused with fresh ACSF of the same composition as above described at a rate of ∼1.5 ml/min. Slices were humidified with a mixed gas consisting of 95% O_2_ and 5% CO_2_ at a constant temperature of 30.0 ± 0.5^o^C.

### Stimulation and Recordings

**FIGURE 1:**
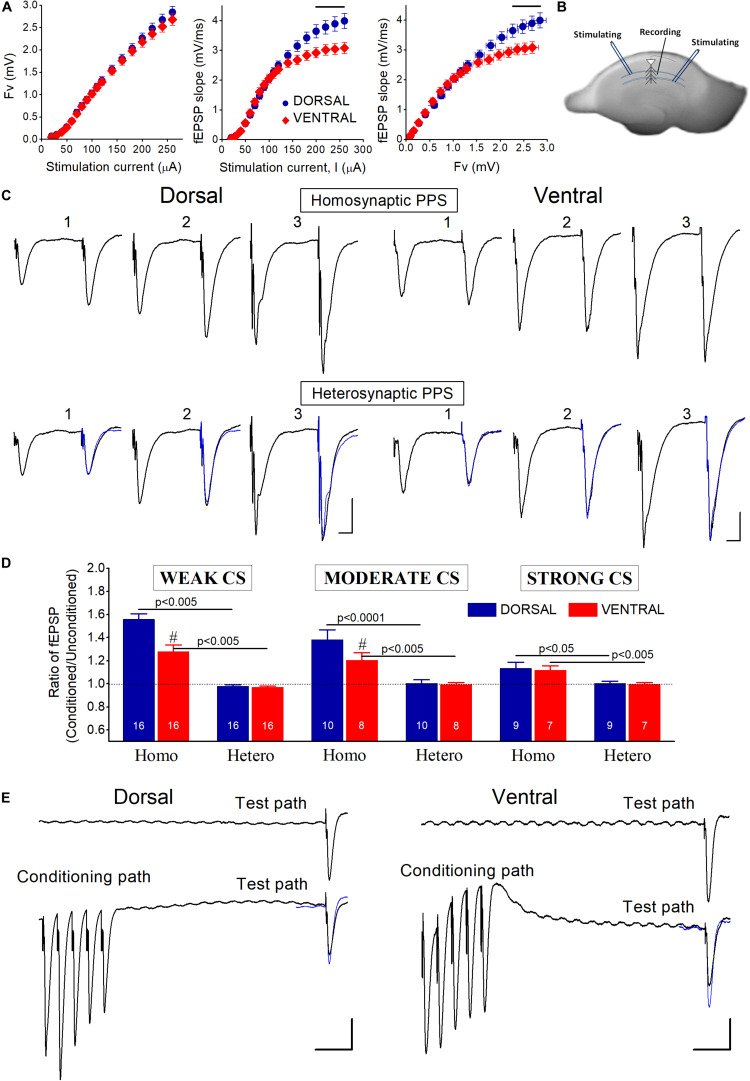
**(A)** Baseline input-output curves of evoked field potentials in the dorsal and the ventral hippocampus. fEPSPs evoked by relatively strong stimulation (≥200 μA), corresponding to large fiber volleys (>2 mV), were significantly larger in the dorsal than in the ventral hippocampus (horizontal lines above data, *p* < 0.05). Data were collected from 83 dorsal and 61 ventral slices obtained from 40 rats. **(B)** Photograph of a transverse hippocampal slice in which is depicted the method of electrical stimulation and recording used to study transient heterosynaptic depression (tHSD) in the CA1 hippocampal field. Two stimulation electrodes were positioned at stratum radiatum on either side of a recording electrode in a way to excite two different sets of presynaptic fibers converging to CA1 pyramidal neurons. One of the inputs was used to condition the response of a test input. The sites of conditioning and test pathways relative to recording electrode randomly alternated between slices. **(C)** Example traces of fEPSPs evoked by paired-pulse stimulation (PPS) of either the same pathway (upper panel, Homosynaptic PPS) or two different pathways (lower panel, Heterosynaptic PPS), in a dorsal and a ventral hippocampal slice. Responses of conditioning and conditioned (test) pathways were evoked by stimulation current of weak (1), moderate (2), and strong (3) intensity. In heterosynaptic PPS panel, superimposed to the conditioned responses are the (unconditioned) responses evoked by the first stimulus in homosynaptic PPS (traces in blue) for comparison sake. The interval between the two pulses in either stimulation configuration (i.e., homosynaptic or heterosynaptic) was 50 ms. Calibration bars: 0.5 mV and 10 ms. The independence of the two pathways was confirmed by the absence of interaction (i.e., facilitation or depression) between conditioning and conditioned responses in the heterosynaptic PPS configuration. On the contrary, homosynaptic PPS produced facilitation of conditioned responses. **(D)** Collective data of the ratio Conditioned fEPSP/Unconditioned fEPSP in homosynaptic (Homo) and heterosynaptic (Hetero) PPS, applied with weak, moderate, and strong conditioning stimulation (CS) current intensity. The number of slices used in each condition is indicated at the bottom of columns. Diesis (#) on top of columns indicate statistically significant difference between the dorsal and the ventral hippocampus (independent *t*-test, *p* < 0.005). Homosynaptic PPS produced synaptic facilitation. On the contrary, heterosynaptic PPS did not significantly affect test responses. **(E)** Examples of tHSD of a test input (Test path) produced by a high-frequency burst (50 Hz) delivered to a distinct set of adjacent fibers (Conditioning path). Upper traces represent responses of the test input before the application of the conditioning burst stimulation, while traces on the bottom represent responses to heterosynaptic stimulation at 300 ms. Calibration bars: 0.5 mV and 50 ms. In the heterosynaptic condition, unconditioned responses of the test pathway (traces in blue) are superimposed to the conditioned responses for comparison. Note that the conditioning burst stimulation produces suppression of fEPSP evoked by stimulation of the test pathway. All artifacts in trace recordings are truncated for clarity.

Electrophysiological recordings started at 1.5−2.0 h after the placement of slices in the recording chamber. Recordings of evoked field potentials consisting of fiber volley (Fv) and field excitatory postsynaptic potentials (fEPSP) in CA1 region were made from the middle of stratum radiatum following electrical stimulation of Schaffer collaterals. We positioned stimulation and recording electrodes in the middle of the stratum radiatum both in the transverse and the radial axis, and, more particularly, 250 and 300−350 μm from the pyramidal cell layer in dorsal and ventral slices, respectively. Considering that the length of stratum radiatum in the middle hippocampus, which is assumed to have similar histological characteristics with the dorsal hippocampus is about 500 μm (Ishizuka et al., 1995), and the length of a CA1 pyramidal cell is about 25−30% higher in the ventral than in the dorsal hippocampus (Dougherty et al., 2012), we assumed that the length of stratum radiatum in dorsal and ventral hippocampal slices is roughly 500 and 600−650 μm, respectively. For recordings we used carbon fiber electrodes (diameter 7 μm, Kation Scientific, Minneapolis, MN, United States), and for stimulation we used a bipolar platinum/iridium electrode (25 μm diameter, at an inter-wire distance of 100 μm, World Precision Instruments, United States). The distance between stimulating and recording electrodes was about 350 μm. Stimulation consisted of electrical current pulses with amplitude of 10−300 μA and a fixed duration of 100 μs. We delivered baseline stimulation every 30 s. Input-output curves between intensity of stimulation current and synaptic response were systematically made in every slice (Figure 1A). Only slices which displayed stable Fv and fEPSP for at least 10 min under fixed stimulation intensity were selected for further experimentation.

We studied transient heterosynaptic depression (tHSD) by applying a brief high-frequency burst (the conditioning stimulation, CS) to a set of afferent hippocampal fibers and observed the depression which is produced in a test (conditioned) response evoked by a single-pulse stimulation of a naive set of adjacent excitatory afferent inputs (Isaacson et al., 1993; Molyneaux and Hasselmo, 2002; Chandler et al., 2003; Zhang et al., 2003; Serrano et al., 2006; Andersson et al., 2007). This pattern of CS resembles physiological neuronal activity in the hippocampus (Lisman, 1997; Buhl and Buzsaki, 2005). More specifically, we placed stimulating electrodes in stratum radiatum of CA1 field, on opposite site of a recording electrode to stimulate two overlapping but independent sets of fibers. We randomly alternated the sides of conditioning and test stimulations between slices, so in some experiments the conditioning stimulus was given at the side of subiculum and in other experiments was applied to the site toward CA3 field. In order to avoid contamination of heterosynaptic depression with homosynaptic effects, we examined the effect of paired-pulse stimulation (PPS). Specifically, we paired single shock stimulation of one pathway with single shock stimulation of the other pathway, using an inter-pulse interval (IPI) of 50 ms. We studied tHSD by applying a burst of five pulses at 50 Hz on one pathway and a single pulse of electrical current to the other pathway (Figure 1B). We adjusted the current intensity of the conditioned stimulus to evoke a subthreshold fEPSP (see Results). We examined the duration of tHSD by varying the interval between the conditioning and test stimulus between 50 and 1700 ms. We repeated the heterosynaptic stimulation protocol three times using three different current intensities of the conditioning stimulus producing an fEPSP with an amplitude of 1 mV (weak conditioning stimulus), 2 mV (moderate conditioning stimulus) and maximum amplitude (strong CS), respectively. Also, we constructed input-output curves between conditioning and test responses by using a moderate CS intensity and a varying amplitude of test stimulation. We also studied the time-course of paired-pulse facilitation (PPF) under control and pharmacological conditions, using a varying IPI (IPIs), from 20 to 1000 ms. We should note here that although fEPSPs that we used to study excitatory synaptic transmission represent mostly postsynaptic depolarizations, however, they are compound synaptic potentials involving monosynaptic excitation as well as monosynaptic and disynaptic (feed-forward) inhibition; furthermore, fEPSPs evoked by relatively high stimulus intensities may also contain polysynaptic components due to anterograde activation of CA1 pyramidal cells and consequent activation of feed-back inhibition, as well as reverberation of CA3 cell excitation, which could then result in CA1 synaptic activation.

### Data Processing and Analysis

Field potentials were amplified 500 times and band-pass filtered at 0.5 Hz − 2 kHz using a Neurolog amplifier (Digitimer Limited, United Kingdom). Signal was digitized at 10 kHz and stored in a computer disk using the CED 1401-plus interface and the Signal6 software (Cambridge Electronic Design, Cambridge, United Kingdom) for off-line analysis. We quantified tHSD as the ratio or the percent change between the conditioned and unconditioned response, i.e., conditioned fEPSP/unconditioned fEPSP or ((conditioned fEPSP − unconditioned fEPSP)/ unconditioned fEPSP)^∗^100. Similarly, we quantified PPF as the ratio between the second and the first response induced by PPS, i.e., fEPSP2/fEPSP1 or ((fEPSP2- fEPSP1)/fEPSP1)^∗^100. Also, changes in tHSD or PPF between different experimental/pharmacological conditions may be expressed by the percent change in the ratio between conditioned (or fEPSP2) and unconditioned response (or fEPSP1) (it is specified in the corresponding text). The fast rising and the slower falling phase of fEPSP were distinctly quantified by the slope and the decay constant “τ,” respectively. In particular, the slope of initial rising phase of fEPSP (fEPSP_slope_) was measured at a time window about 1 ms-wide, after the occurrence of the presynaptic fiber volley; the time constant of fEPSP decay (fEPSP_τ_) was measured by the time required for fEPSP to decrease by 63% from its maximum amplitude. The size of fEPSP_slope_ is an accurate indication of the strength of synaptic activation, while fEPSP_τ_ closely reflects changes in the slow decaying phase of fEPSP. Fv was quantified by its amplitude measured by the difference between the baseline and the peak negative voltage.

### Drugs

The following drugs were used: the selective antagonists of GABA_B_R 3-[[(3,4-Dichlorophenyl)methyl]amino]propyl] diethoxymethyl)phosphinic acid (CGP 52432, 10 μM) and 3-aminopropyl)(cyclohexylmethyl)phosphinic acid (CGP 46381, 50 μM); the selective agonist of GABA_B_Rs baclofen; the competitive selective antagonist of NMDA receptor 3-((R)-2-Carboxypiperazin-4-yl)-propyl-1-phosphonic acid (CPP, 10 μM); the selective antagonist of adenosine A_1_R 8-Cyclopentyl-1,3-dipropylxanthine (DPCPX, 150 nM), the blocker of GABA_A_R picrotoxin (PTX, 5 μM) and the blocker of L-VGCCs nimodipine (20 μM). DPCPX, CGP46381, CGP52432, baclofen, CPP and nimodipine were purchased from Tocris Cookson Ltd., United Kingdom; PTX was obtained from Sigma-Aldrich, Germany. Drugs were first prepared as stock solutions and then dissolved in standard medium and bath applied to the tissue. Stock solutions of baclofen, CGP52432 and CPP were prepared in distilled water, whereas stock solutions of PTX, DPCPX and nimodipine were prepared in dimethyl-sulfoxide (DMSO) at a concentration that when diluted for bath application the final volume of DMSO was lower than 0.005%.

### Statistical Analysis

The following tests were used for statistical comparisons: paired and independent *t*-tests, multivariate general linear model (MANOVA), one-way analysis of variance (ANOVA), non-linear regression analysis and bivariate correlation analysis. The IBM SPSS and GraphPad Prism 8 software packages were used for statistical analyses. The values in the text and figures express mean ± SEM. Values are expressed as mean ± S.E.M and “n” throughout the text indicates the number of slices used in the analysis.

## Results

### tHSD Is Stronger in the Ventral Compared With the Dorsal Hippocampus

In this study we made field recordings from 194 dorsal and 168 ventral hippocampal slices prepared from 118 adult rats. Input-output curves showed that presynaptic fiber volley (Fv) was similar in dorsal and ventral slices along the entire range of stimulation current intensities used (50−260 μA) (Figure 1A). However, fEPSPs evoked by strong presynaptic activation corresponding to stimulation current ≥200 μA and Fv > 2 mV, were significantly larger in the dorsal than in the ventral hippocampus (independent *t*-test, *p* < 0.05).

Considering that at CA3-CA1 synapses both time-course and mechanisms involved in heterosynaptic depression depend on the intensity of afferent fiber activation (Isaacson et al., 1993; Manzoni et al., 1994; Scanziani et al., 1996; Zhang et al., 2003; Serrano et al., 2006; Andersson et al., 2007), we studied the effects of heterosynaptic stimulation using three different levels of CS intensity: weak, moderate, and strong CS, that evoked an fEPSP_slope_ of 0.48 ± 0.02 mV/ms, 1.1 ± 0.04 mV/ms, and 2.0 ± 0.1 mV/ms, respectively. We adjusted the stimulation current intensity to evoke a test fEPSP_slope_ of 0.57 ± 0.03 mV/ms in dorsal (*n* = 67) and 0.55 ± 0.02 mV/ms in ventral slices (*n* = 59). Independence of the two stimulated pathways was ascertain by applying a PPS paradigm in which two fast succeeding stimuli were delivered to either the same stimulating electrode or the two different stimulating electrodes, at an IPI of 50 ms (Sastry et al., 1986; Grover and Teyler, 1993a; Zhang et al., 2003; Andersson et al., 2007). We proceeded to study tHSD when heterosynaptic PPS produced no change on conditioned synaptic responses, as opposed to facilitation produced by PPS delivered to an individual pathway. We examined independence of conditioning and test pathways using weak, moderate, and strong stimulation intensities. Homosynaptic PPS produced significant facilitation of fEPSP_slope_ in both dorsal and ventral hippocampal slices, at all stimulation current intensities (paired *t*-test in each hippocampal segment and stimulation current intensity, *p* < 0.05) (Figures 1C,D). Furthermore, homosynaptic PPF was significantly greater in dorsal than in ventral hippocampal synapses when produced by weak and moderate, but not strong, stimulation intensity (independent *t*-test, *p* < 0.05), as previously described (Papatheodoropoulos and Kostopoulos, 2000b; Maruki et al., 2001; Papatheodoropoulos, 2015b; Milior et al., 2016; Babiec et al., 2017). Heterosynaptic PPS, however, produced no significant change in fEPSP_slope_ at any intensity of CS, in either kind of hippocampal slices (Figures 1C,D), verifying the independence of the two pathways.

**FIGURE 2:**
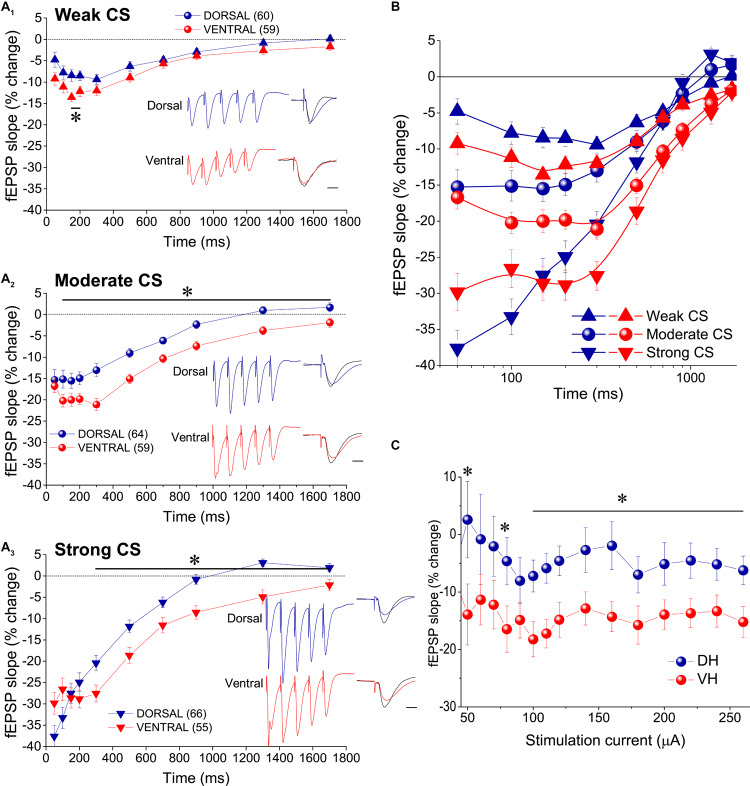
tHSD is stronger and longer in the ventral compared with the dorsal hippocampus. **(A)** Diagrams presenting the time course of tHSD induced in the dorsal and the ventral hippocampus by weak **(A_1_)**, moderate **(A_2_)**, or strong **(A_3_)** intensity of CS. Horizontal bars with an asterisk indicate the interval of statistically significant differences in tHSD between the dorsal and the ventral hippocampus, at *p* < 0.05 (paired *t*-test). Insert graphs represent example fEPSP trace recordings; a 150 ms-long interval between conditioning and test response is omitted for clarity. Traces in black denote control responses at the conditioned path. Calibration bars: 1 mV, 10 ms. Artifacts are truncated. **(B)** Time courses of tHSD induced by the three intensities of CS are shown for the dorsal and the ventral hippocampus for comparison. **(C)** Dorsal-ventral difference in tHSD produced by a CS of moderate intensity on a test response evoked by a stimulation intensity of variable size. The inter-stimulation interval was set at 300 ms.

In order to study tHSD in CA1 field we applied a conditioning brief tetanus consisting of a five pulse-burst at 50 Hz to a set of Schaffer collaterals (conditioning path) and we observed the depression induced in a different test response evoked by stimulation of a naive set of excitatory afferent inputs (test path). We examined the time course of tHSD by varying the interval between conditioning and test stimulus, from 50 to 1700 ms. We found that burst stimulation of the conditioning pathway reliably induced a transient reduction of fEPSP_slope_ in the test pathway in either kind of hippocampal slices. Furthermore, the magnitude of tHSD depended on the intensity of CS; tHSD was stronger at IPIs of 50−300 ms and fainted at longer intervals, of 1300−1700 ms (Figure 2). Importantly, tHSD was stronger and lasted longer in ventral compared with dorsal hippocampal slices. More specifically, the weak CS produced a significant reduction in the test fEPSP_slope_ at IPIs of 50−900 ms in dorsal slices and 50−700 in ventral slices (paired *t*-test between control and test fEPSP_slope_ in each interval and hippocampal segment, *p* < 0.05) (Figures 2A_1_,B). CS of moderate strength significantly reduced fEPSP_slope_ at 50−900 ms in dorsal and 50−1700 ms in ventral slices (paired *t*-test, between control and test fEPSP_slope_, *p* < 0.05) (Figures 2A_2_,B). The depression of the test fEPSP_slope_ was significantly greater in ventral than in dorsal slices for IPIs 150−200 ms when induced by weak intensity of CS, and for IPIs 100−1700 ms when induced by moderate intensity of CS (independent *t*-test between dorsal and ventral slices, for each intensity of CS and IPIs, *p* < 0.05) (Figure 2A_2_). Strong CS induced tHSD at 50−700 ms in dorsal slices and 50−1300 ms in ventral slices (paired *t*-test between control and test fEPSP_slope_ in each interval and hippocampal segment, *p* < 0.05) (Figures 2A_3_,B). tHSD induced by strong CS was significantly stronger in the ventral than in the dorsal hippocampus at IPIs of 300−1300 ms (independent *t*-test, *p* < 0.05). The dorsal-ventral difference in tHSD was also evident when comparing the effect of a conditioning stimulus of moderate strength on test response of variable size, at an IPI of 300 ms (Figure 2C). These results showed that tHSD is stronger and longer in the ventral than in the dorsal hippocampus.

### PPF Increases During tHSD

**FIGURE 3:**
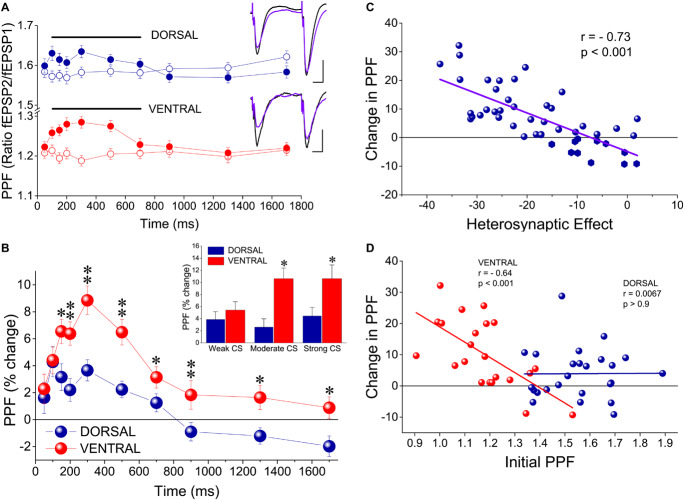
tHSD is associated with a greater increase in paired-pulse facilitation (PPF) in the ventral compared with the dorsal hippocampus. **(A)** PPF before (open symbols) and during tHSD (filled symbols) in dorsal and ventral hippocampal slices. PPF is shown as the ratio between the second and the first fEPSP evoked by PPS of the test pathway at an IPI of 50 ms. Examples of recordings illustrating PPF at the test pathway before (traces in black) and after burst stimulation at the conditioning path are also shown. Calibration bars: 0.5 mV, 10 ms. Artifacts are truncated. Data were obtained from all three intensities of CS. Horizontal bars indicate statistically significant differences (paired *t*-test). The level of significance for the dorsal hippocampus is *p* < 0.05 (200 ms, 700 ms) and *p* < 0.005 (100−150 ms, 300−500 ms), and for the ventral hippocampus *p* < 0.001 (100−500 ms) and *p* < 0.01 (700 ms). **(B)** Comparison of PPF increase between the dorsal and the ventral hippocampus. Asterisks indicate statistically significant differences between the two hippocampal segments at *p < 0.05, **p < 0.005. Data were obtained from all three intensities of CS. Insert: Comparison of PPF increase produced by heterosynaptic CS at 300 ms between the dorsal and the ventral hippocampus. Data are shown separately for the three intensities of CS. Asterisk denote statistically significant difference between the two hippocampal segments at *p* < 0.05. **(C)** Diagram of PPF changes induced by heterosynaptic CS plotted against corresponding scores of tHSD. Changes in PPF significantly correlated with the amount of tHSD (bivariate correlation analysis). **(D)** Diagram of change in PPF plotted against initial PPF. Data are shown separately for the dorsal and the ventral hippocampus. Changes in PPF inversely correlated with (relatively low) initial amount of PPF in the ventral but not the dorsal hippocampus (bivariate correlation analysis). Symbols represent mean value of individual slices calculated by pooling all three intensities of CS for an IPI of 300 ms.

It has been previously proposed that tHSD results principally from a reduction in glutamate release, thereby tHSD is associated with an increase in synaptic facilitation as revealed by a PPS paradigm (Molyneaux and Hasselmo, 2002). Accordingly, we examined whether tHSD is accompanied by a change in PPF by applying two stimuli of equal intensity to the test input and observed the effect of heterosynaptic CS. We found that PPF of the test input was significantly increased when preceded by a burst delivered to the conditioning input. Specifically, PPF significantly increased in both the dorsal and the ventral hippocampus at intervals of 100−700 ms (paired *t*-test of test responses before and after CS, *p* < 0.05 in the dorsal, and *p* < 0.01 in the ventral hippocampus) (Figure 3A). Furthermore, the increase in PPF was higher in the ventral than in the dorsal hippocampus at a range of IPIs from 150 to 500 ms (independent *t*-test; level of significance from *p* < 0.05 to *p* < 0.001; see the legend of Figure 3 for more statistics) (Figure 3B). Additionally, we found a significant dorsal-ventral difference for moderate and strong, but not weak, CS (insert in Figure 3B). The change in PPF induced by heterosynaptic stimulation was positively correlated with tHSD, i.e., higher scores of tHSD associated with higher changes in PPF (Figure 3C). Interestingly, the changes in PPF induced by heterosynaptic stimulation inversely correlated with the initial amount of PPF in the ventral but not the dorsal hippocampus (Figure 3D). These data suggested that presynaptic mechanisms contribute to tHSD.

### tHSD Depends on GABA_B_R in Both Hippocampal Segments

**FIGURE 4:**
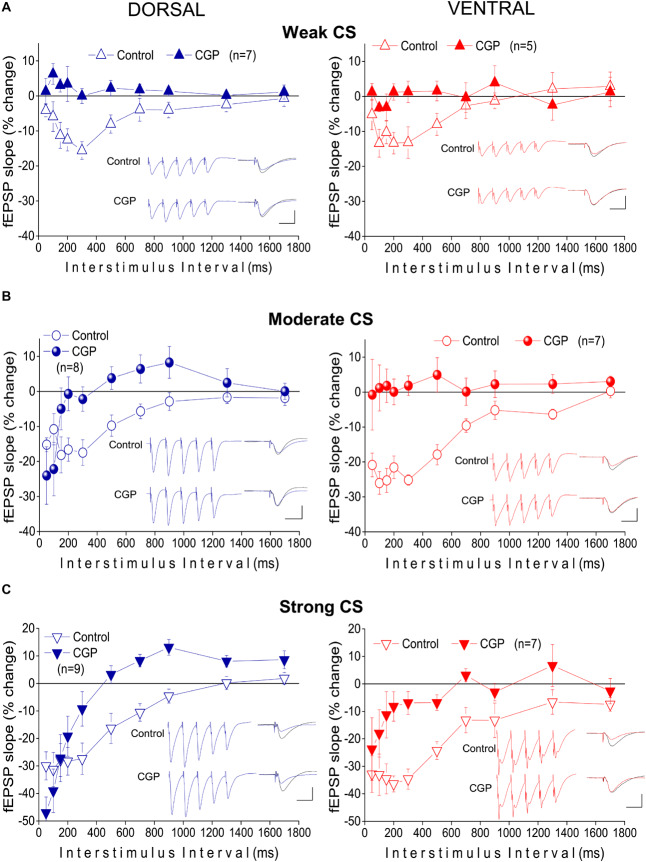
Heterosynaptic depression depends on GABA_B_Rs in both the dorsal and the ventral hippocampus. Time courses of tHSD induced under normal conditions (open symbols) or blockade of GABA_B_Rs by their selective antagonists CGP 52432 (10 μM) or CGP 46381 (50 μM) (filled symbols), in the dorsal and the ventral hippocampus. Three intensities of CS, weak **(A)**, moderate **(B)**, or strong **(C)** were used to induce tHSD. Numbers into parenthesis indicate the number of slices used. Insets are examples of fEPSP recordings for an inter-stimulus interval of 200 ms; a 150 ms-long interval between conditioning and test response is omitted for clarity reasons. Traces in black denote control responses of the conditioned path. Calibration bars: 1 mV, 10 ms. Artifacts are truncated. Note that blockade of GABA_B_Rs by CGP eliminates tHSD induced by weak CS, in both hippocampal segments, and tHSD induced by moderate CS in the ventral hippocampus. However, significant tHSD induced by moderate CS under blockade of GABA_B_Rs remained in the dorsal hippocampus, at 50−100 ms. Also, significant tHSD induced by strong CS under blockade of GABA_B_Rs remained in the dorsal (50−200 ms) and the ventral hippocampus (50−100 ms).

The previous results showed that under the specified experimental conditions short-lasting tHSD in CA1 field is stronger and last longer in the ventral compared with the dorsal hippocampus. In the CA1 hippocampal field, all forms of heterosynaptic depression including fast or tHSD has been proposed to depend on heterosynaptic GABA_B_Rs (Isaacson et al., 1993; Serrano et al., 2006; Andersson et al., 2007). According to a proposed mechanism, the GABA released by the conditioning high-frequency burst stimulation spill over into neighboring excitatory synapses and depress glutamate release by activating presynaptic GABA_B_ heteroreceptors (Isaacson et al., 1993; Covelo and Araque, 2016). To determine whether tHSD involved activation of GABA_B_Rs, we applied the selective GABA_B_R antagonists CGP 52432 (10 μM) or CGP 46381 (50 μM) to dorsal and ventral slices. We found that blockade of GABA_B_Rs completely abolished tHSD induced by weak CS in both the dorsal and the ventral hippocampus (paired *t*-test between control and drug conditions, in each hippocampal segment, *p* < 0.05) (Figure 4A). Also, blockade of GABA_B_Rs eliminated tHSD induced by moderate CS at IPIs ≥ 150 ms in the dorsal, and IPIs ≥ 50 ms in the ventral hippocampus (paired *t*-test in each hippocampal segment, *p* < 0.05). Thus, in the dorsal but not the ventral hippocampus, CS of moderate intensity continued to induce significant depression of the test response at 50−100 ms under blockade of GABA_B_Rs (Figure 4B). Furthermore, blockade of GABA_B_Rs abolished depression induced by strong CS at ≥ 300 ms in the dorsal and ≥ 150 ms in the ventral hippocampus (paired *t*-test, *p* < 0.05) (Figure 4C). The depression of fEPSP_slope_ produced by strong CS in the dorsal hippocampus remained significant at 50−200 ms (paired *t*-test, *p* < 0.05). Therefore, in the dorsal hippocampus, pharmacological blockade of GABA_B_Rs failed to reduce tHSD that was induced at relatively short IPIs by moderate or strong CS. Furthermore, under blockade of GABA_B_Rs, the test fEPSP_slope_ in the dorsal hippocampus significantly increased by strong CS at IPIs ≥ 700 ms (paired *t*-test, *p* < 0.05). Blockade of GABA_B_Rs significantly increased baseline fEPSP_slope_ in dorsal (by 10.1 ± 2.3%, *n* = 22, *p* < 0.01) but not ventral hippocampal slices (5.9 ± 2.5%, *n* = 15, *p* > 0.05). fEPSP_slope_ was adjusted to pre-drug levels before performing heterosynaptic stimulation. These results suggested that GABA_B_Rs are involved in tHSD in both hippocampal segments, with an increased role in the ventral hippocampus, especially at higher intensities of CS.

### A_1_Rs, GABA_A_Rs, and L-VGCCs Differently Contribute to tHSD in the Dorsal and the Ventral Hippocampus

**FIGURE 5:**
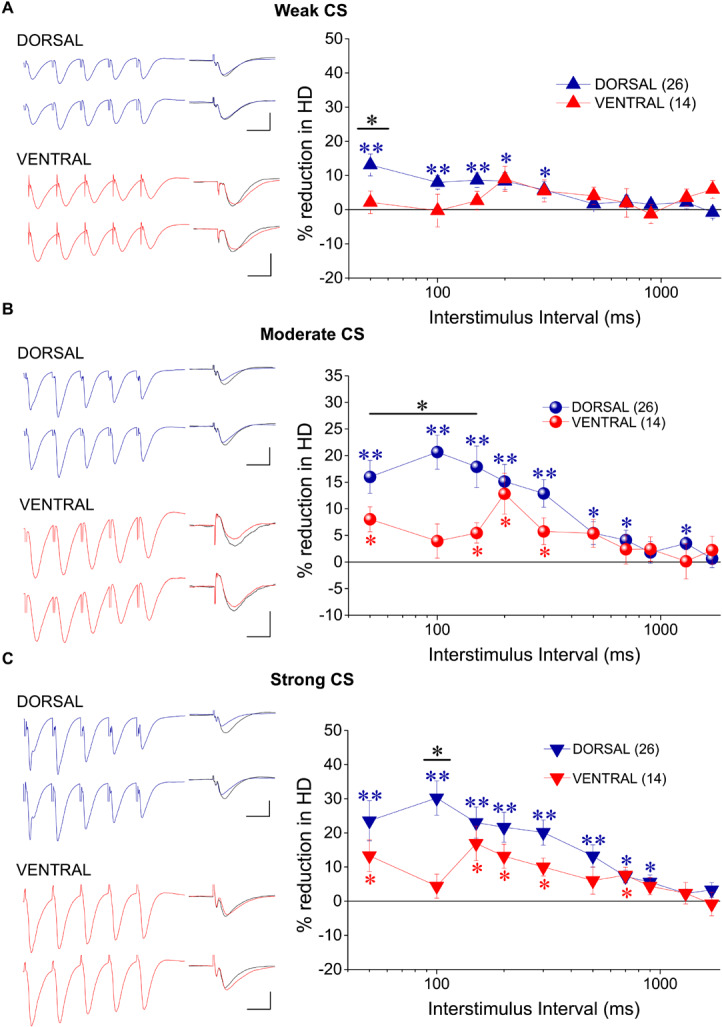
tHSD involves activation of adenosine A_1_Rs more in dorsal than in ventral hippocampus. Examples of fEPSP recordings (at an inter-stimulus interval of 200 ms) and collective results are shown in the left and the right panel, respectively. The reducing effects of the antagonist of A_1_Rs DPCPX (150 nM) on tHSD are presented as a percent change of tHSD between normal and drug conditions. The drug effects are shown for weak **(A)**, moderate **(B)**, and strong intensity of CS **(C)**. In example traces, a 150 ms-long interval between conditioning and test response is omitted for clarity reasons. Traces in black denote control responses at the conditioned path. Calibration bars: 1 mV, 10 ms. Artifacts are truncated. Numbers into parenthesis indicate the number of slices used. Asterisks above (dorsal hippocampus) or below symbols (ventral hippocampus) denote statistically significant differences between normal and drug conditions, at *p < 0.05 and **p < 0.001 (paired *t*-test). Horizontal bars denote statistically significant differences between the dorsal and the ventral hippocampus, at *p < 0.05 and **p < 0.001 (independent *t*-test). For additional statistical tests (ANOVA, MANOVA) see main text.

It has been previously shown that the mechanism through which GABA_B_Rs induce tHSD may involve activity of adenosine A_1_ receptors (A_1_Rs) located on glutamatergic terminals that inhibit glutamate release (Zhang et al., 2003; Serrano et al., 2006). Therefore, we investigated whether A_1_Rs participate to tHSD by applying heterosynaptic stimulation in the presence of DPCPX (150 μM), in 26 dorsal and 14 ventral hippocampal slices. We found that blockade of A_1_Rs consistently reduced tHSD more in dorsal than in ventral hippocampal slices (Figure 5). In the dorsal hippocampus, DPCPX significantly reduced tHSD induced by CS of weak (ANOVA, *F* = 4.23, *p* < 0.001), moderate (ANOVA, *F* = 7.95, *p* < 0.001), and strong intensity (ANOVA, *F* = 6.8, *p* < 0.001) (for more statistical details, see legend of Figure 5). Regarding the duration of tHSD produced by weak, moderate, and strong CS in the dorsal hippocampus, we observed statistically significant drug-induced changes at 50−300, 50−700, and 50−1300 ms, respectively (paired *t*-test, *p* < 0.001 − *p* < 0.05; the exact level of significance in given in Figure 5). In the ventral hippocampus, DPCPX reduced tHSD induced by moderate and strong CS, but not weak CS, at a limited range of IPIs (50, 150−300 ms, paired *t*-test, *p* < 0.05; see Figure 5). The effects of DPCPX were significantly greater in the dorsal than in the ventral hippocampus for moderate (MANOVA, *F* = 14.52, *p* < 0.001), and strong CS (MANOVA, *F* = 15.22, *p* < 0.001). DPCPX significantly increased baseline fEPSP_slope_ in both dorsal (30.21 ± 5.13%, *n* = 23, *p* < 0.001) and ventral hippocampal slices (23.35 ± 9.32%, *n* = 15, *p* < 0.05) similarly (independent *t*-test, *p* > 0.5), as also previously observed (Reis et al., 2019). fEPSP_slope_ was adjusted to pre-drug levels before performing heterosynaptic stimulation.

**FIGURE 6:**
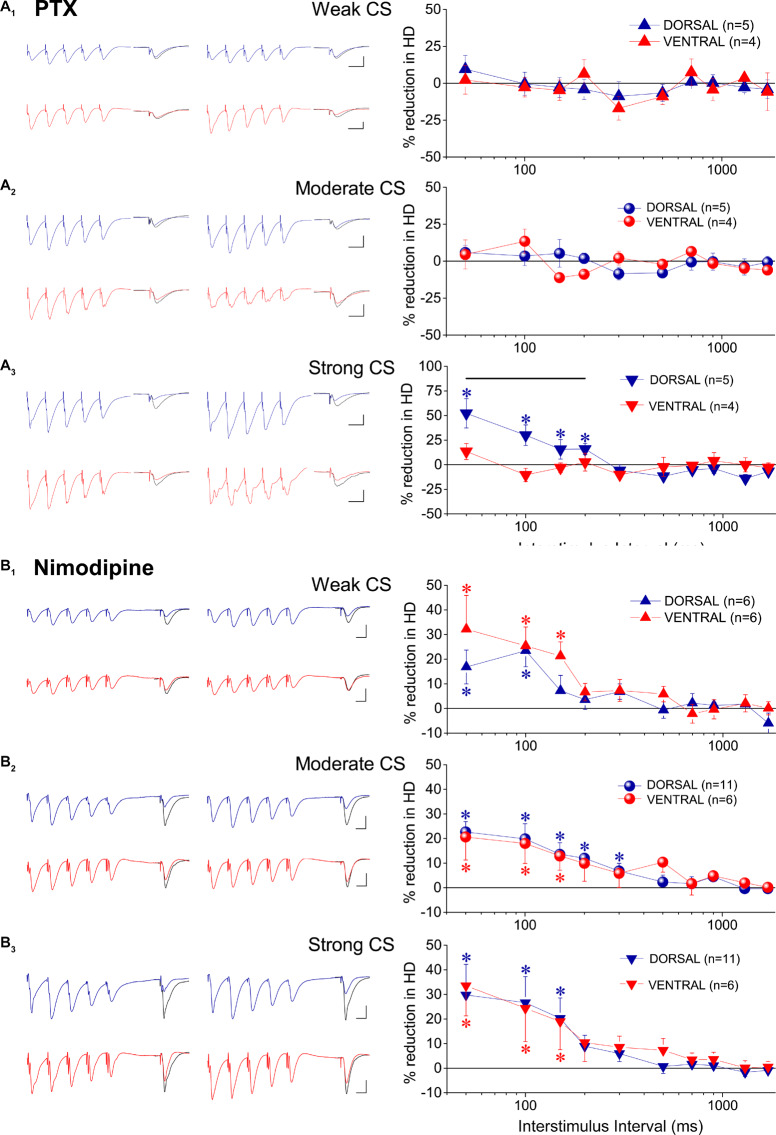
GABA_A_Rs and L-VGCCs are differently involved in tHSD in the dorsal and the ventral hippocampus. The effects of blockade of GABA_A_Rs by 5 μM PTX, and the effects of blockade of L-VGCCs by nimodipine are shown in **(A,B)**, respectively. Examples of fEPSP recordings (at an inter-stimulus interval of 100 ms and 50 ms for PTX and nimodipine, respectively) and collective results (percent change of tHSD between normal and drug conditions) are shown in the left and the right panel, respectively. In example traces for PTX, a 60 ms-long interval between conditioning and test response is omitted for clarity. Traces in black denote control responses at the conditioned path. Calibration bars: 1 mV, 10 ms. All artifacts are truncated. Numbers into parenthesis indicate the number of slices used. Asterisks above or below symbols denote statistically significant differences between normal and drug conditions, at *p < 0.05 (paired *t*-test). Horizontal bar denotes statistically significant differences between the dorsal and the ventral hippocampus, at *p* < 0.05 (independent *t*-test). For additional statistical tests see main text. Note that blockade of GABA_A_Rs significantly reduced tHSD induced by strong CS at short intervals, only in the dorsal hippocampus. Blockade of L-VGCCs reduced tHSD induced by all intensities of CS, in both hippocampal segments.

Considering that in addition of presynaptic GABA_B_ heteroreceptors, postsynaptic GABA_A_ receptors may be involved in reducing conditioned responses (Davies and Collingridge, 1996), we examined the effects of PTX on tHSD in five dorsal and four ventral hippocampal slices. We used a low concentration of PTX (5 μM) to avoid possible confounding effects of PTX through actions on serotonergic or nicotinic receptors (Liu et al., 1994; Erkkila et al., 2004; Das and Dillon, 2005; Thompson, 2013). We found that PTX significantly reduced tHSD induced by strong CS in the dorsal hippocampus (ANOVA, *F* = 7.4, *p* < 0.001). This effect was seen at relatively short IPIs (50−200 ms) (Figure 6A). We observed no significant effects of PTX on tHSD induced in the ventral hippocampus at any intensity of CS or IPI (Figure 6A). PTX increased baseline fEPSP_slope_ in both dorsal (by 13.7 ± 2.5%, paired *t*-test, *p* < 0.01) and ventral hippocampal slices (by 11.3 ± 1.1%, paired *t*-test, *p* < 0.01). Thus, we adjusted fEPSP_slope_ to pre-drug levels before performing heterosynaptic stimulation.

Postsynaptic depolarization induced by conditioning burst-stimulation may recruit L-VGCCs in the dendrites of CA1 pyramidal cells (Andreasen and Lambert, 1995; Geier et al., 2011), Activation of L-VGCCs by conditioning input may then reduce conditioned fEPSPs, located near conditioning input, by shunting excitatory synaptic currents (Häusser et al., 2001). Following application of the blocker of L-VGCCs nimodipine we observed a significant reduction in tHSD induced by weak, moderate, and strong CS, in both hippocampal segments (Figure 6B). Specifically, nimodipine applied in the dorsal hippocampus produced reduction in tHSD induced by weak (*F* = 3.6, *p* < 0.005), moderate (*F* = 4.6, *p* < 0.001), and strong intensity of CS (*F* = 3.7, *p* < 0.005) (ANOVA). Similarly, application of nimodipine in the ventral hippocampus reduced tHSD induced by weak (*F* = 3.2, *p* < 0.05), moderate (*F* = 2.0, *p* < 0.05), and strong intensity of CS (*F* = 2.1, *p* < 0.05) (ANOVA). Drug-reduced reductions in tHSD were higher at shorter IPIs (see results from paired *t*-test in Figure 6B). Also, nimodipine significantly increased the baseline fEPSP_slope_ in the dorsal (10.6 ± 2.5%, *n* = 11, paired *t*-test, *p* < 0.005) but not the ventral hippocampus (2.0 ± 3.2%, *n* = 6, paired *t*-test, *p* > 0.5). We adjusted fEPSP_slope_ to pre-drug levels before performing heterosynaptic stimulation in the presence of nimodipine.

### GABA_B_R Suppresses fEPSP_Slope_ and Enhances fEPSP_τ_ Differently in the Two Hippocampal Segments

**FIGURE 7:**
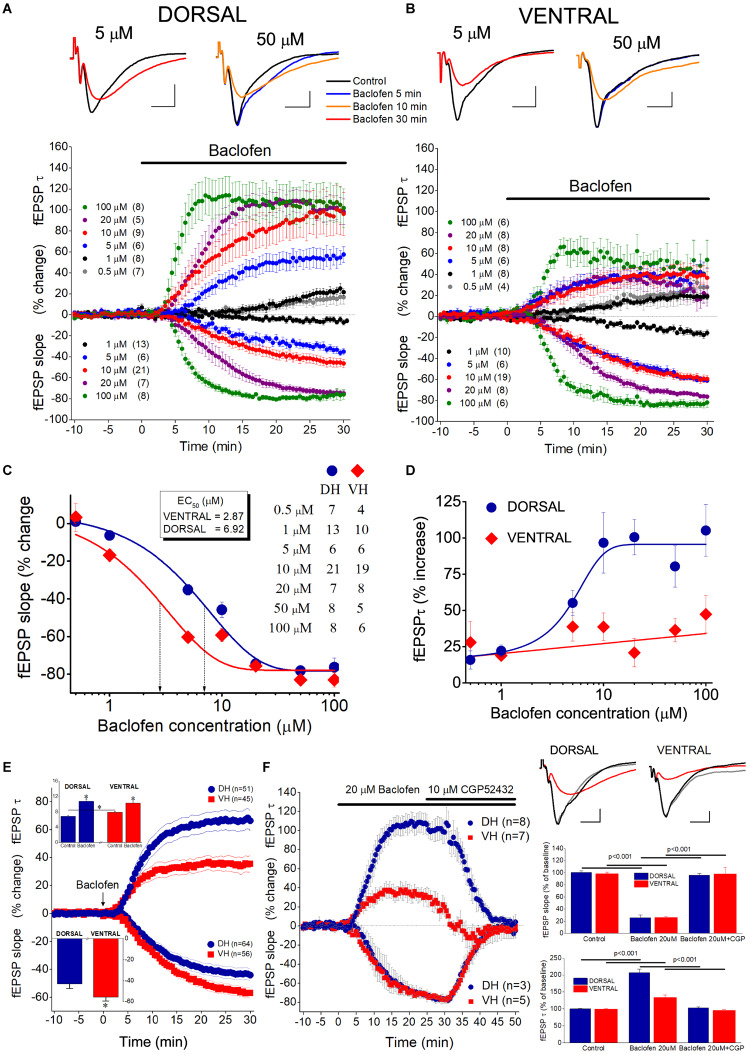
Activation of GABA_B_Rs by baclofen suppresses fEPSP_slope_ more in the ventral than in the dorsal hippocampus and prolongs fEPSP_τ_ more in the dorsal than in the ventral hippocampus. Collective time courses of percent change of fEPSP_slope_ and fEPSP_τ_ illustrating the action of baclofen in the dorsal hippocampus and the ventral hippocampus are shown in panels **(A,B)**, respectively. In collective diagrams, values shown below and above zero correspond to changes in fEPSP_slope_ and fEPSP_τ_, respectively. The number of slices studied is shown into parenthesis in collective graph legends. Data for 0.5 μM (fEPSP_slope_) and 50 μM (fEPSP_slope_ and fEPSP_τ_) are not shown in collective graphs for clarity reasons. Representative examples of fEPSP traces, shown on the top of graphs, were collected under control conditions and under 5 or 50 μM baclofen. Calibration bars: 0.5 mV, 5 ms. Note that 5 μM baclofen suppresses fEPSP_slope_ more in the ventral than in the dorsal hippocampus and at the same time prolongs the decay phase of fEPSP in the dorsal but not the ventral hippocampus. Also note that a 5 min application of 50 μM baclofen induces an increase in fEPSP decay phase in the dorsal but not the ventral hippocampus, i.e., before any change in fEPSP_slope_ occurs (blue trace, baclofen 5 min). **(C)** Concentration-effect curves for baclofen actions on fEPSP_slope_. Curves were constructed using the Boltzmann function. The number of slices used is indicated in the legend. Dotted arrowed lines indicate EC_50_ values for the ventral (VH, 2.57 μM) and the dorsal hippocampus (DH, 5.98 μM). The two curves differ significantly (non-linear regression analysis, *p* < 0.0001). **(D)** Concentration-effect relationship of baclofen actions on fEPSP_τ_. The number of slices used for all baclofen concentrations, but 50 μM, is shown in panel **(B)**; the effects of 50 μM baclofen were studied in eight dorsal and five ventral hippocampal slices. **(E)** Graph of cumulative time courses of baclofen-induced changes in fEPSP_slope_ and fEPSP_τ_ (suppression and enhancement, respectively) in the dorsal and the ventral hippocampus, constructed by pooling data from all baclofen concentrations. Lines on both sides of symbols indicate SEM. The number of slices studied in given into parenthesis. Arrow depicts the start of baclofen application (time = 0), which continues for the next 30 min. The inset graph on the top-left of panel shows the mean values of fEPSP_τ_ under control and drug conditions. Asterisks indicate statistically significant differences at *p* < 0.05 (paired *t*-test and independent *t*-test were used for comparisons inside and between hippocampal segments, respectively). Note that under control conditions the value of fEPSP_τ_ is higher in the ventral than in dorsal hippocampus; following baclofen application the value of fEPSP_τ_ becomes similar in the two hippocampal segments. The inset graph on the bottom-left of the panel shows that baclofen-induced suppression of fEPSP_slope_ was significantly higher in the ventral than in dorsal hippocampus (asterisk, independent *t*-test, *p* < 0.05). **(F)** Are shown time course diagrams (graph on the left), example traces of fEPSP recordings (upper-right panel) and aggregate data under control and drug conditions (lower-right graphs) illustrating that 10 μM CGP52432 fully reversed the effects of 20 μM baclofen on fEPSP_slope_ and fEPSP_τ_ in the dorsal and the ventral hippocampus. Horizontal lines indicate statistically significant differences; the level of significance is also given. Calibration bars: 0.5 mV, 5 ms.

To investigate the effects of GABA_B_Rs’ activation by the endogenous GABA on excitatory synaptic transmission, comparatively in the dorsal and the ventral hippocampus, we applied the agonist of GABA_B_R baclofen in dorsal and ventral hippocampal slices and we measured drug effects on fast and slow components of fEPSP. Specifically, we measured fEPSP_slope_ that represents the fast component of excitatory synaptic transmission, and the decay time constant “τ” of fEPSP (fEPSP_τ_) that represents the time required for a fEPSP to fall to 37% of its maximum amplitude and quantifies the slow component of synaptic transmission. We applied baclofen at a wide range of concentrations that covered extracellular GABA levels in the hippocampus (Wilson et al., 1996; Çavuş et al., 2016) and saturating drug concentrations (Pfrieger et al., 1994; Labouèbe et al., 2007). Also, in an effort to reduce a possible effect of GABA_B_R desensitization (Froestl, 2010; Turecek et al., 2014), we applied only one drug concentration in individual slices. We adjusted stimulation current intensity to evoke a half-maximum fEPSP_slope_, in dorsal (1.41 ± 0.06 mV/ms, *n* = 61) and ventral (1.36 ± 0.09, *n* = 50) hippocampal slices. We found that baclofen produced robust and concentration-dependent changes in fEPSP_slope_ in both the dorsal (ANOVA, *F* = 40.1, *p* < 0.0001) and the ventral hippocampus (*F* = 65.1, *p* < 0.0001). Furthermore, the suppressive effect of baclofen significantly differed between the two hippocampal segments, as demonstrated by comparing the concentration-response curves (Figure 7C). Specifically, following fitting Boltzmann function to data we found a significantly higher suppression of fEPSP_slope_ in the ventral than in the dorsal hippocampus; non-linear regression analysis, *F*(DFn, DFd) = 9.023 (3, 122), *p* < 0.0001. EC_50_ values were 2.87 μM for the dorsal and 6.92 μM for the ventral hippocampus. These results suggested that the potency of baclofen to suppress excitatory synaptic transmission is higher in the ventral compared with the dorsal hippocampus.

Baclofen, in addition to suppress fEPSP_slope_, produced a significant increase in fEPSP_τ_ in both hippocampal segments (Figures 7A,B). Furthermore, the enhancement of fEPSP_τ_ was depended on the drug concentration in the dorsal hippocampus (ANOVA, *F* = 7.2, *p* < 0.0001) but not the ventral hippocampus (ANOVA, *F* = 1.6, *p* > 0.1). In dorsal slices this effect was remarkably fast, and at large drug concentrations the prolongation of fEPSP (i.e., the increase in fEPSP_τ_) preceded the reduction in fEPSP_slope_, as it is evident in the time courses of drug effects (see Figure 7A). Strikingly, fEPSP_τ_ increased despite a robust reduction in fEPSP_slope_, and baclofen concentrations (0.5−1 μM) that did not significantly affect fEPSP_slope_ produced a considerable increase in fEPSP_τ_ (15.8 ± 6.3% and 22.3 ± 3.9% at 0.5 and 1 μM, respectively, paired *t*-test, *p* < 0.05). In the ventral hippocampus the maximum baclofen-induced increase in fEPSP_τ_ (47.4 ± 12.9%) was about half of the maximum drug effect observed in the dorsal hippocampus (105.2 ± 17.8%) and the effects of baclofen on fEPSP_τ_ across all baclofen concentrations significantly differed between the dorsal and the ventral hippocampus [non-linear regression analysis, *F*(DFn, DFd) = 15.25 (3, 100), *p* < 0.0001] (Figure 7D). The dorsal-ventral difference in baclofen effects was also observed after pooling all drug concentrations together, for fEPSP_slope_ (-43 ± 4.2% vs. -55.7 ± 3.7% in the dorsal and ventral hippocampus, respectively; independent *t*-test, *p* < 0.05) and fEPSP_τ_ (67.7 ± 7.2% vs. 35.0 ± 5.8% in the dorsal and ventral hippocampus, respectively, independent *t*-test, *p* < 0.001) (Figure 7E). The effects of baclofen on fEPSP_slope_ and fEPSP_τ_ were fully reversed following application of the specific antagonist of GABA_B_ receptors CGP52432 (10 μM), in both dorsal and ventral hippocampal slices (Figure 7F).

**FIGURE 8:**
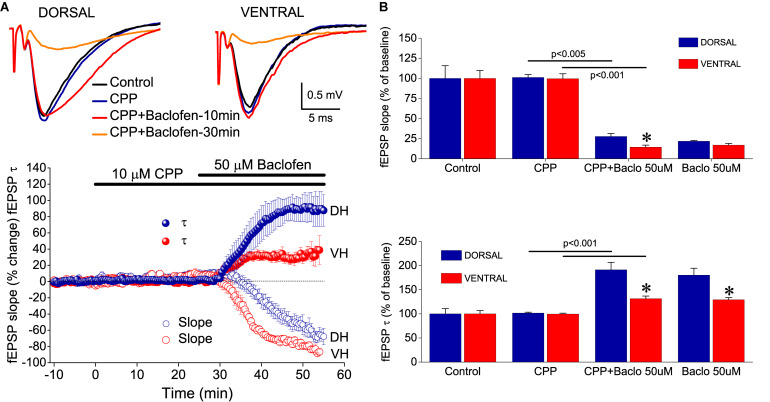
NMDA receptors are not involved in baclofen-induced effects on fEPSP. **(A)** Example traces of fEPSP recordings (upper panel) and time-courses (graph on the bottom) of percent changes in fEPSP_slope_ and fEPSP_τ_ induced by 50 μM baclofen under blockade of NMDA receptors by CPP. **(B)** Cumulative data obtained during application of CPP (25 min) and CPP + baclofen (30 min) are shown for fEPSP_τ_ and fEPSP_slope_ (upper and lower graph, respectively). Statistically significant differences between experimental conditions are shown by horizontal lines and the level of significance is given above lines. Asterisks indicate statistically significant differences between the dorsal and the ventral hippocampus, at *p* < 0.05. CPP did not prevent the effects of baclofen on fEPSP_τ_ or fEPSP_slope_ in either hippocampal segment.

The effects of baclofen on fEPSP_slope_, but not fEPSP_τ_, most probably result from the activation of GABA_B_ heteroreceptors located on glutamatergic terminals. The baclofen effects on fEPSP_τ_, however, appeared to be consistent with an activation of GABA_B_ autoreceptors. GABA_B_ autoreceptors suppress GABA release and reduce postsynaptic inhibition (Thompson and Gahwiler, 1992; Bowery, 1993), thereby allowing generation of an EPSP with increased duration (Nathan and Lambert, 1991; Davies and Collingridge, 1996). Interestingly, suppression of fast postsynaptic inhibition facilitates the activation of NMDA receptors (Dingledine et al., 1986), and NMDA receptors may significantly contribute to the decay phase of fEPSP in CA1 hippocampal field (Andreasen et al., 1989; Papatheodoropoulos, 2015b). Therefore, we hypothesized that NMDA receptors may contribute to the baclofen-induced increase in fEPSP_τ_. In order to test this hypothesis, we applied CPP in seven dorsal and seven ventral hippocampal slices before the application of 50 μM baclofen. We found that blockade of NMDA receptors by CPP did not prevent the enhancing effect of baclofen on fEPSP_τ_ (Figure 8). Also, CPP did not significantly change basal fEPSP_τ_ and fEPSP_slope_ and did not significantly affect the suppressive effect of baclofen on fEPSP_slope_. These results suggested that baclofen increased fEPSP_τ_ by indirectly enhancing the activation of non-NMDA glutamatergic receptors.

### GABA_B_R Increases Synaptic Facilitation in the Ventral and Dorsal Hippocampus Similarly

**FIGURE 9:**
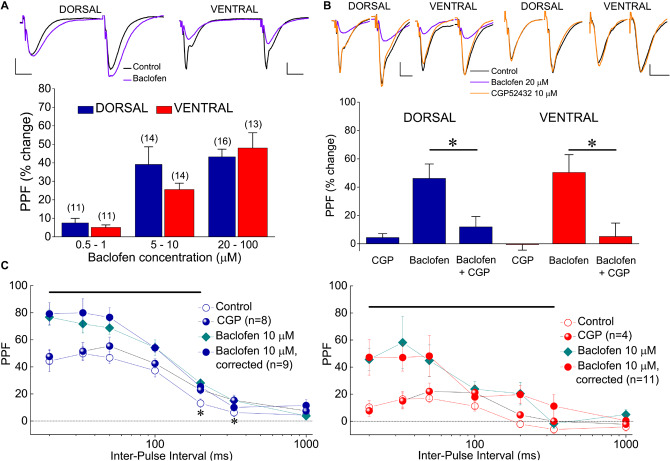
Baclofen enhances PPF of fEPSP_slope_. **(A)** Cumulative data of the effects of various baclofen concentrations on PPF are shown for the dorsal and the ventral hippocampus; example traces obtained before (traces in black) and during application of 10 μM baclofen (traces in violet) in a dorsal and a ventral slice are shown on the top of the graph. In the example of the dorsal hippocampus, similar unconditioned fEPSP_slope_ under control and drug conditions were selected for comparison reasons. Drug concentrations are segregated into three groups: 0.5−1, 5−10, and 20–100 μM. PPF is expressed as the percent change of the ratio fEPSP2/fEPSP1 observed under baclofen with respect to control conditions. The number of slices used in each condition is indicated into parenthesis on the top of each bar. Baclofen significantly increased PPF in both hippocampal segments (ANOVA, *F* = 4.8, *p* < 0.005 in dorsal and *F* = 5.6, *p* < 0.001 in ventral hippocampus). **(B)** The antagonist of GABA_B_R CGP52432 (10 μM) reversed the increase of PPF induced by 20 μM baclofen in four dorsal and four ventral slices (asterisks indicate statistically significant difference with respect to control values, at *p* < 0.05, paired *t*-test). Blockade of GABA_B_Rs under basal conditions did not significantly affect PPF in either dorsal (*n* = 8) or ventral (*n* = 4) hippocampal slices. In all cases PPF was induced at 50 ms. Respective examples are shown on the top of the graph. **(C)** PPF plotted as a function of IPI in dorsal (left) and ventral (right) under control conditions, application of CGP52432, and application of baclofen with or without adjustment of fEPSP_slope_ to control levels. Horizontal bars denote statistically significant differences between control and baclofen-corrected conditions (paired *t*-test, *p* < 0.05). Also, asterisks in the left graph indicate that application CGP52432 significantly increased PPF in dorsal slices.

Presynaptic receptors that control transmitter release can also modulate short-term synaptic plasticity, which is typically represented by PPF. PPF is inversely correlated with changes in transmitter release and manipulations that reduce transmitter release enhance PPF (Manabe et al., 1993). Therefore, given the higher baclofen-induced suppression of excitatory synaptic transmission in the ventral compared with the dorsal hippocampus, we asked whether baclofen could affect PPF differently in the two hippocampal segments. We examined the effects of varying concentrations of baclofen on PPF (measured as the percentage of fEPSP2/fEPSP1 ratio) at an IPI of 50 ms in both kinds of hippocampal slices. We found that baclofen significantly increased PPF in a concentration-dependent manner in both hippocampal segments, similarly (Figure 9A). These effects reversed upon application of 10 μM CGP 52432 (Figure 9B). We also characterized the effect of 10 μM baclofen on the time course of PPF at a wide range of IPIs, from 20 to 1000 ms. We found that baclofen significantly increased PPF in both the dorsal and the ventral hippocampus. Specifically, baclofen significantly increased PPF in the dorsal hippocampus for IPIs up to 200 ms (paired *t*-test, *p* < 0.05) and in the ventral hippocampus for IPIs up to 333 ms (paired *t*-test, *p* < 0.05) (Figure 9C). It is known that PPF depends on the size of the conditioning fEPSP (i.e., fEPSP1) (Manabe et al., 1993; Papatheodoropoulos, 2015b), which is shown here that is robustly reduced by baclofen. Therefore, in the condition of baclofen, we examined PPF also after adjusting the fEPSP_slope_ to the control levels to exclude the case that the baclofen-induced increase in PPF was secondary to the reduction of fEPSP1. We found that PPF after fEPSP_slope_ correction was similar with that measured before correction (Figure 9C, Baclofen 10 μM, correction). Furthermore, blockade of GABA_B_R under basal conditions did not significantly affect PPF in either hippocampal segment (Figure 9C, CGP).

## Discussion

The main findings of this study are the following: (a) tHSD induced by paired stimulation of two independent inputs to stratum radiatum of CA1 field is stronger in the ventral compared with the dorsal hippocampus; (b) Induction of tHSD depends on several mechanisms, with a predominant participation of GABA_B_Rs and A1Rs in the dorsal hippocampus and GABA_B_Rs in the ventral hippocampus; (c) Exogenous activation of GABA_B_Rs by baclofen, suppresses the initial fast component of excitatory synaptic transmission more efficiently in the ventral than in the dorsal hippocampus, and enhances the late slow component of excitatory transmission more efficiently in the dorsal than in the ventral hippocampus; (d) Exogenous, but not endogenous tonic, activation of GABA_B_Rs enhances PPF in the dorsal and the ventral hippocampus similarly.

### Dorsal-Ventral Differences in Heterosynaptic Interactions

In keeping with previous studies (Gustafsson et al., 1989; Isaacson et al., 1993; Molyneaux and Hasselmo, 2002; Chandler et al., 2003; Zhang et al., 2003; Andersson et al., 2007; Guetg et al., 2009) we found that short burst of high-frequency stimulation induces tHSD that lasts less than 2 s. Furthermore, we show for the first time that tHSD has a greater magnitude and lasts longer in the ventral than in the dorsal hippocampus. Several mechanisms, have been implicated in heterosynaptic depression in hippocampus (Covelo and Araque, 2016). A commonly accepted general mechanism that is proposed for the induction of tHSD includes the release of GABA from interneurons and activation of inhibitory presynaptic GABA_B_ heteroreceptors on glutamate terminals, resulting thus in suppression of glutamate release and reduction of excitatory synaptic transmission (Isaacson et al., 1993; Molyneaux and Hasselmo, 2002; Chandler et al., 2003; Serrano et al., 2006; Andersson et al., 2007; Guetg et al., 2009). Furthermore, adenosine released during high-frequency CS may also participate to tHSD via activation of presynaptic A_1_Rs on glutamate terminals, thereby contributing to reduction of excitatory glutamate transmission (Grover and Teyler, 1993b; Zhang et al., 2003). In keeping with these proposed mechanisms, we found that tHSD induced in both the dorsal and the ventral hippocampus involves activation of GABA_B_Rs and A1Rs. However, in this study we found that GABA_B_Rs and A1Rs do not contribute, similarly, to the dorsal and the ventral hippocampus (see Supplementary Table 1 for a schematically illustrated contribution of various mechanisms to tHSD in the two hippocampal segments). GABA_B_Rs and A1Rs appear to have an overall similar participation in the induction of tHSD in the dorsal hippocampus, although the participation of GABA_B_Rs increases toward weak stimulation intensities and the relative contribution of A1Rs increases toward strong intensities (Supplementary Table 1). In the ventral hippocampus, GABA_B_Rs appear to play a more prominent role than A1Rs, which participate at relatively strong, and not weak, CS intensities. Seemingly, A1Rs are involved more in the dorsal than in the ventral hippocampus, presumably reflecting the increased expression of these receptors in the dorsal CA1 hippocampal field (Lee et al., 1983; Reis et al., 2019). The putative action of presynaptic mechanisms in tHSD is corroborated by the fact that tHSD is associate with an enhancement in PPF since reduction in presynaptic transmitter release can produce an increase in the magnitude of PPF (Manabe et al., 1993; Dumas and Foster, 1998b). However, additional, presumably postsynaptic, GABA_B_R-dependent mechanisms may participate in tHSD, contributing to dorsal-ventral diversification. For instance, strong CS delivered in the dorsal and not the ventral hippocampus under blockade of GABA_B_Rs uncovered a facilitation of the test response at IPIs ≥ 700 ms (Figure 5C), suggesting that under control conditions postsynaptic GABA_B_Rs may participate in suppressing conditioned responses in the dorsal hippocampus, thereby contributing to dorsal-ventral difference in tHSD induced by strong CS. Interestingly, previous observations have suggested that postsynaptic GABA_B_R-mediated hyperpolarizations are greater in the dorsal than in the ventral hippocampal pyramidal cells (Papatheodoropoulos et al., 2002) and GABA_B_Rs appear to control postsynaptic depolarizations in CA1 pyramidal cells more efficiently in the dorsal than in the ventral hippocampus (Papatheodoropoulos, 2015a).

In addition, here we provide the first evidence for the involvement of GABA_A_Rs and L-VGCCs in tHSD, and we show that GABA_A_Rs have an increased contribution to tHSD in the dorsal hippocampus, while L-VGCCs have a similar participation in the two hippocampal segments. The increased contribution of GABA_A_Rs to tHSD in the dorsal hippocampus is consistent with the increased GABA_A_R-dependent inhibition in this hippocampus segment (Petrides et al., 2007). The reduction of tHSD by nimodipine may involve blockade of L-VGCCs (Moyer and Disterhoft, 1994), and reduction of the slow afterhyperpolarization (Power et al., 2002; Kaczorowski, 2011), which can be enhanced by activation of L-VGCCs (Thibault and Landfield, 1996). Voltage-dependent potassium channels, activation of which can inhibit postsynaptic depolarizations, may also contribute to the effects of nimodipine on tHSD since nimodipine can block these channels in some preparations (Caro et al., 2011). Overall, the present evidence suggests that tHSD in the CA1 hippocampal field presents different properties between the dorsal and the ventral hippocampus. Furthermore, a multitude of presynaptic and postsynaptic mechanisms contribute in shaping tHSD, and a distinct pattern of these mechanism participation occurs between the two segments of the hippocampus.

Presynaptic inhibition, in addition to simply restrict transmitter release, may play important functional roles in brain networks. For example, heterosynaptic depression may increase contrast between activated and non-activated synapses (Dunwiddie and Lynch, 1978), or between background activity and responses to specific external stimuli in brain networks (Frerking and Ohliger-Frerking, 2006), and may also enhance the importance of a relatively strong input in a field (Frerking and Ohliger-Frerking, 2006). Also, heterosynaptic depression may increase and restrict synaptic saturation (Lynch et al., 1977). Accordingly, we hypothesize that the observed dorsal-ventral differences in tHSD may reflect an important facet of diversified organization along hippocampus, concerning input segregation and input selectivity. GABA_B_Rs appear to play a prominent role in tHSD. Given that GABA_B_Rs require pooling of synaptically released GABA to be activated (Scanziani, 2000; Kulik et al., 2003, 2006), GABA_B_Rs may contribute to tHSD during transients in extracellular GABA that can occur under conditions of enhanced and/or synchronous activity of GABAergic neurons, and they may serve to detect this activity in local networks (Osten et al., 2007). Interestingly, sharp wave and ripples, an endogenous network activity of the hippocampus that importantly assist in the process of memory consolidation (Buzsaki, 2015), reflects synchronized GABAergic cell activity in CA1 local network (Wu et al., 2002; Papatheodoropoulos, 2008), and displays an increased generation in the ventral hippocampus (Kouvaros and Papatheodoropoulos, 2017). Furthermore, during sharp wave-ripples there is a very selective activation of hippocampal CA1 principal cells (Chrobak and Buzsaki, 1996; Papatheodoropoulos, 2008). Hence, we speculate that an increased tHSD in the ventral hippocampus may facilitate sharpening of input segregation and assist the property of input-output selectivity in the functioning of local CA1 circuit on the context of sharp wave-ripples, according to their role as carries of bits of mnemonic information.

### GABA_B_R-Dependent Control of fEPSP_Slope_ and fEPSP_τ_

GABA_B_ receptor is a heterodimeric metabotropic G protein-coupled receptor for GABA with both presynaptic and postsynaptic actions (Jones et al., 1998; Bowery et al., 2002; Bettler et al., 2004). Heterosynaptic GABA_B_Rs on glutamatergic terminals is a powerful mechanism that controls excitatory synaptic transmission in the hippocampus, among other brain regions (Ault and Nadler, 1982; Dutar and Nicoll, 1988a; Thompson and Gahwiler, 1992; Bowery, 1993; Chalifoux and Carter, 2011). Furthermore, the suppressive effects of baclofen on excitatory transmission at CA3-CA1 synapses is well known (Ault and Nadler, 1982; Dutar and Nicoll, 1988b; Thompson and Gahwiler, 1992). However, this study investigated for the first time the effects of GABA_B_Rs’ activation by an exogenous agonist on excitatory synaptic transmission, comparatively between the dorsal and the ventral hippocampus. The present results suggest that GABA_B_Rs control the fast component of glutamatergic transmission more efficiently in the ventral than in the dorsal hippocampus; furthermore, somewhat surprisingly, we found that baclofen robustly increased the slow decaying phase of fEPSP, more in the dorsal than in the ventral hippocampus. Thus, it appears that activation of GABA_B_R has a dual effect on the excitatory synaptic transmission in the CA1 hippocampal field, restricting the fast component and promoting the slow component of glutamatergic transmission. Furthermore, the efficiency of this dual effect is reverse in the two segments of the hippocampus.

The dual effects of baclofen on fEPSP_slope_ and fEPSP_τ_, may have different mechanistic interpretations. On the one side, the baclofen-induced suppression of fEPSP_slope_ could safely be explained in terms of action of GABA_B_ heteroreceptor on glutamate terminals, as previously established (Ault and Nadler, 1982; Dutar and Nicoll, 1988b; Thompson and Gahwiler, 1992; Bowery, 1993; Vizi and Kiss, 1998; Ulrich and Bettler, 2007; Chalifoux and Carter, 2011). The enhancing effect of baclofen on fEPSP_τ_, on the other side, may result from an interaction between presynaptic and postsynaptic mechanisms. Specifically, the slow decaying phase of fEPSP, represented by fEPSP_τ_, temporally coincides with the period of fast postsynaptic GABA_A_R-mediated inhibition (Andersen et al., 1964; Alger and Nicoll, 1982), which can very effectively control the duration of postsynaptic depolarizations (Nathan and Lambert, 1991; Davies and Collingridge, 1996). It is then obvious that the waveform of fEPSP reflects a compound synaptic potential shaped not only by monosynaptically produced depolarizations, but also by mononynaptic and disynaptic (feed-forward/feed-back) inhibition. At high stimulus intensities may also contribute polysynaptic components resulted from reverberation of CA3 cell excitation that can result in CA1 synaptic activation. Accordingly, a reduction in GABA_A_R-mediated postsynaptic inhibition may contribute to the enhancement of fEPSP_τ_. Indeed, activation of GABA_B_ autoreceptors on GABAergic terminals leads to a reduction in GABA release followed by suppression in postsynaptic inhibition (Davies et al., 1990, 1991; Nathan and Lambert, 1991; Thompson and Gahwiler, 1992; Bowery, 1993; Davies and Collingridge, 1996). Therefore, we hypothesize that the baclofen-induced increase in fEPSP_τ_ could result from an increased activity of GABA_B_ autoreceptors and a consequent reduction in postsynaptic fast inhibition. The differences we observed in the effects of baclofen between the dorsal and the ventral hippocampus could not easily be interpreted by the recently reported increased expression of GABA_B_Rs in the CA1 stratum radiatum of the ventral hippocampus (Dubovyk and Manahan-Vaughan, 2018), since although we found a lower EC_50_ value for baclofen in the ventral than in the dorsal hippocampus, the maximum drug action did not differ between the two hippocampal segments, as could be suggested by the increased quantitative GABA_B_R expression in the ventral hippocampus. In addition, expression profiles found in the above-cited study could refer either to GABA_B_ heteroreceptors or to GABA_B_ autoreceptors, as well as to postsynaptic GABA_B_Rs. Alternatively, the dorsal-ventral difference in baclofen-induced effects on fEPSP could be related to the wide molecular and functional heterogeneity of GABA_B_Rs (Ulrich and Bettler, 2007; Schwenk et al., 2010).

Presynaptic mechanisms that control the release of neurotransmitter play a crucial role in regulating the balance between excitation and inhibition in local neuronal circuits and thus determining the dynamic state of excitability required for normal activity in brain networks (Haider et al., 2006). Several lines of evidence suggest that an increased excitability characterizes the intrinsic neuronal network of the ventral hippocampus, presumably representing a constitutive property of this segment of hippocampus that can reliably assist to its normal functions (Papatheodoropoulos, 2018). For instance, pyramidal cells in the ventral hippocampus have an increased intrinsic excitability (Dougherty et al., 2012; Honigsperger et al., 2015; Malik et al., 2016; Milior et al., 2016), reduced GABA_A_ receptor-mediated synaptic inhibition (Papatheodoropoulos et al., 2002; Petrides et al., 2007; Maggio and Segal, 2009), and increased NMDA receptor-dependent activity (Papatheodoropoulos et al., 2005). Nevertheless, under some conditions the ventral hippocampus displays an increased tendency to fall into a hyperexcitability state that results in pathologic activity, as shown both *in vivo* (Spencer et al., 1984; Quigg et al., 1997) and *in vitro* (Bragdon et al., 1986; Borck and Jefferys, 1999; Mikroulis and Psarropoulou, 2012; Papatheodoropoulos, 2015a). However, the requirements for long-term stability and normal function, suggest that some mechanisms intrinsic to the local network may function to counterbalance the tendency of the ventral hippocampus for increased excitability. For instance, ventral pyramidal cells display decreased network-driven spontaneous activity (Kouvaros and Papatheodoropoulos, 2017), and increased level of calcium-activated potassium channels of SK-type (Babiec et al., 2017). Most GABA_B_Rs, including presynaptic ones, are located distant from sites of GABA release and presumably require increased level of GABA to be activated (Davies and Collingridge, 1996; Scanziani, 2000; Lopez-Bendito et al., 2004; Kulik et al., 2006). Increased levels of GABA can be achieved under conditions of increased network activity that may endow the risk of runaway excitation (Kulik et al., 2003). Interestingly, in the present study we observed a greater suppressive effect of baclofen on fast excitatory transmission in the ventral compared with the dorsal hippocampus, at low micromolar baclofen concentrations, similar with those of GABA that occur under conditions of intense network activity (Roth and Draguhn, 2012). Furthermore, the enhancing effects of baclofen on the slow decaying phase of fEPSP were limited in the ventral compared with the dorsal hippocampus. Thus, activation of GABA_B_Rs under conditions of increased network activity may represent a homeostatic mechanism that contributes to keep network activity in the ventral hippocampus within a physiological range.

### Effects of GABA_B_Rs on Short-Term Plasticity

Because changes in the probability that a transmitter is released by a presynaptic terminal affect the amount of transmitter released by subsequent afferent activity, one of the functional roles that presynaptic inhibition of transmitter release can play is the modulation of short-term synaptic plasticity (Hennig, 2013; Mukunda and Narayanan, 2017; Cheng et al., 2018). Short-term synaptic plasticity crucially influences the dynamics of local neuronal network activity and can play important roles in information processing performed by brain networks, including information filtering and input diversification (Silver, 2010; Jackman and Regehr, 2017). Decreased ability for short-term synaptic plasticity can directly influence the ability of a synapse to sustain bursts of presynaptic activity and to forward neural information in local brain networks (Lisman, 1997; Koutsoumpa and Papatheodoropoulos, 2019). In addition, a reduced short-term synaptic plasticity may crucially hamper the ability for induction of long-term plasticity, especially under conditions of long bouts of afferent activity (Kouvaros and Papatheodoropoulos, 2016b; Papaleonidopoulos et al., 2018). Among other mechanisms, short-term synaptic plasticity critically depends on the properties of transmitter release, including transmitter release probability; for instance, PPF is inversely related to the probability of transmitter release at a given synapse (Dobrunz and Stevens, 1997; Fioravante and Regehr, 2011; Regehr, 2012).

In keeping with previous results (Papatheodoropoulos and Kostopoulos, 2000b; Maruki et al., 2001; Papatheodoropoulos, 2015b; Kouvaros and Papatheodoropoulos, 2016a; Moschovos and Papatheodoropoulos, 2016; Babiec et al., 2017; Dubovyk and Manahan-Vaughan, 2018), we found that PPF in CA hippocampal fields is higher in dorsal than in ventral hippocampal synapses, an observation that represents one of the most remarkable and established intrinsic differences between the dorsal and the ventral hippocampus. Considering the inverse relationship between PPF and probability of transmitter release, it has been proposed that ventral hippocampal synapses possess an increased transmitter release probability (Papatheodoropoulos and Kostopoulos, 2000b; Papatheodoropoulos, 2015b). It has been shown that experimental manipulations that inhibit transmitter release will impact on synapse with higher than lower probability of transmitter release (Manabe et al., 1993). Indeed, activation of GABA_B_Rs by baclofen increase PPF in hippocampus (Manabe et al., 1993; Dumas and Foster, 1998a; Margrie et al., 2000; Lei and McBain, 2003). Here, we tested the hypothesis of increased transmitter release probability in the ventral hippocampal synapses by asking whether activation of GABA_B_Rs by baclofen will produce a higher enhancement of PPF in the ventral than in the dorsal hippocampus. Contrary to the hypothetical prediction, we found that baclofen produced a similar increment in PPF in dorsal and ventral hippocampus, suggesting that dorsoventral differences in PPF involve complex mechanisms of regulation of transmitter release that may not entirely reside in properties like the probability of transmitter release (Babiec et al., 2017). However, during heterosynaptic stimulation, that represents a more physiologically relevant condition compared with application of an exogenous GABA_B_R agonist, PPF increases significantly more in the ventral than in the dorsal hippocampus (see Figure 3) and the change in PPF induced by heterosynaptic stimulation inversely correlated with initial PPF scores only in the ventral hippocampus, suggesting that basal probability of transmitter release in CA1 excitatory synapses differs between the two hippocampal segments. Importantly, however, PPF did not change in either the dorsal or the ventral hippocampus following blockade of GABA_B_Rs suggesting that GABA_B_Rs do not tonically regulate basal probability of glutamate release in either hippocampal segments which, therefore, may have similar probabilities of transmitter release. Predictively, a more precise determination of the glutamate release probability at the CA3-CA1 synapses in the dorsal and the ventral hippocampus should await future research, engaging several approaches that by necessity include detailed quantal analysis of transmitter release.

## Concluson

Concluding, we argue that the present data show that the excitatory synaptic transmission is differently modulated between the dorsal and the ventral CA1 hippocampal field. tHSD of the excitatory transmission has an increased magnitude and duration in the ventral hippocampus, and depends on multiple mechanisms, which have, however, a different participation in the two segments of the hippocampus. GABA_B_Rs predominate in the ventral hippocampus and both GABA_B_Rs and A1Rs play important roles in the dorsal hippocampus. Activation of GABA_B_Rs by an exogenous agonist controls the initial fast component of excitatory synaptic transmission more in the ventral than in the dorsal hippocampus and enhances the late slow component of excitatory transmission mainly in the dorsal hippocampus. We propose that under conditions of increased neuronal activity (experimentally mimicked by relatively intense heterosynaptic stimulation or baclofen application), input segregation is increased in the ventral hippocampus, while at the same time, the reduction in excitatory transmission may contribute in dampening the, endogenously increased, neuronal excitability of the ventral hippocampus network. Furthermore, the increased GABA_B_R-dependent control of the slow component of glutamatergic transmission in the dorsal hippocampus may serve to gate excitatory input more efficiently in this segment of the hippocampus. The shown differences may provide a means for specialization of information processing in the local neuronal circuits, thereby significantly contributing to functional segregation along hippocampus.

## Data Availability Statement

The raw data supporting the conclusions of this article will be made available by the authors, without undue reservation, to any qualified researcher.

## Ethics Statement

The animal study was reviewed and approved by the Protocol Evaluation Committee of the Department of Medicine of the University of Patras and the Directorate of Veterinary Services of the Achaia Prefecture of Western Greece Region, Papadiamanti 14 and Aretha, 26443, Patras.

## Author Contributions

GT performed the experiments and analyzed the data. CP designed the study, analyzed the data, and wrote and prepared the manuscript.

## Conflict of Interest

The authors declare that the research was conducted in the absence of any commercial or financial relationships that could be construed as a potential conflict of interest.
