# Supplementary material for: Dorsal-Ventral Differences in Modulation of Synaptic Transmission in the Hippocampus
Source: Front Synaptic Neurosci. 2020 Jun 18;12:24. doi: 10.3389/fnsyn.2020.00024 (PMC7316154; doi:10.3389/fnsyn.2020.00024)
Supplement: Supplementary file 1 [file Data_Sheet_1.PDF]

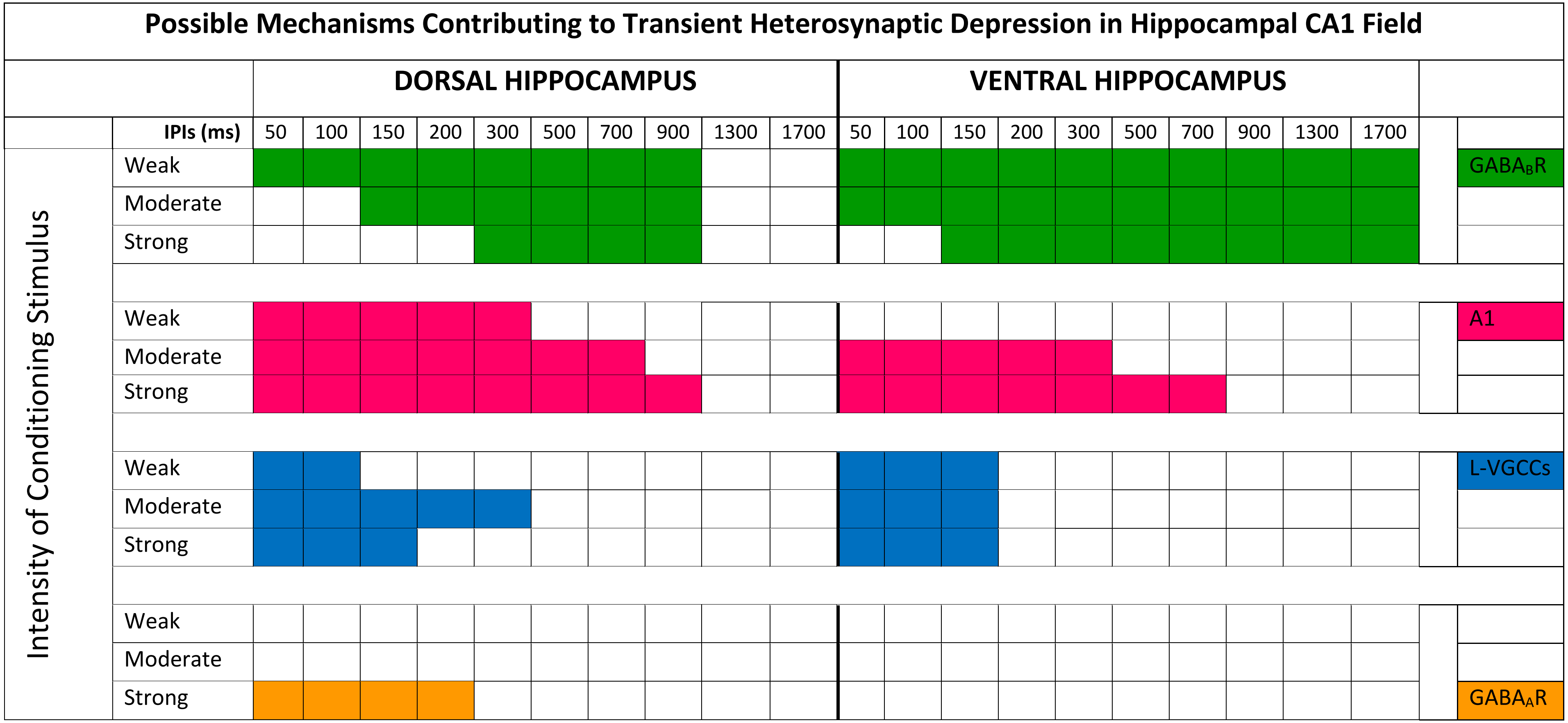

The participation of each identified mechanism, as revealed by drug-induced significant reduction in tHSD, is depicted by a distinct color.
